# Proteomic Study to Survey the CIGB-552 Antitumor Effect

**DOI:** 10.1155/2015/124082

**Published:** 2015-10-20

**Authors:** Arielis Rodríguez-Ulloa, Jeovanis Gil, Yassel Ramos, Lilian Hernández-Álvarez, Lisandra Flores, Brizaida Oliva, Dayana García, Aniel Sánchez-Puente, Alexis Musacchio-Lasa, Jorge Fernández-de-Cossio, Gabriel Padrón, Luis J. González López, Vladimir Besada, Maribel Guerra-Vallespí

**Affiliations:** ^1^Department of Proteomics, Center for Genetic Engineering and Biotechnology, 10600 Havana, Cuba; ^2^Department of Bioinformatics, Center for Genetic Engineering and Biotechnology, 10600 Havana, Cuba; ^3^Pharmaceutical Department, Center for Genetic Engineering and Biotechnology, 10600 Havana, Cuba

## Abstract

CIGB-552 is a cell-penetrating peptide that exerts *in vitro* and *in vivo* antitumor effect on cancer cells. In the present work, the mechanism involved in such anticancer activity was studied using chemical proteomics and expression-based proteomics in culture cancer cell lines. CIGB-552 interacts with at least 55 proteins, as determined by chemical proteomics. A temporal differential proteomics based on iTRAQ quantification method was performed to identify CIGB-552 modulated proteins. The proteomic profile includes 72 differentially expressed proteins in response to CIGB-552 treatment. Proteins related to cell proliferation and apoptosis were identified by both approaches. In line with previous findings, proteomic data revealed that CIGB-552 triggers the inhibition of NF-*κ*B signaling pathway. Furthermore, proteins related to cell invasion were differentially modulated by CIGB-552 treatment suggesting new potentialities of CIGB-552 as anticancer agent. Overall, the current study contributes to a better understanding of the antitumor action mechanism of CIGB-552.

## 1. Introduction

Peptides, owing to their feasible rational design, high specificity, and low toxicity, have become attractive therapeutic agents to treat different diseases [1]. Ongoing advances in cancer therapy include the discovery of peptides with a potent antineoplastic effect [2]. Peptides inhibiting angiogenesis or blocking protein-protein interactions have already been evaluated as potential anticancer agents [3]. Additionally, proapoptotic peptides or peptides functioning as receptor antagonists have been proposed to restrict tumor progression [3].

CIGB-552 is a second-generation peptide derived from the antitumor peptide L-2. Initially, L-2 was identified by screening a peptide library corresponding to the region 32–51 of* Limulus* anti-LPS factor (LALF) [4]. The cytotoxic effect of L-2 was demonstrated on different murine and human tumor cell lines [4]. A transcriptomic study on tumor cells suggested that L-2 could induce apoptosis by modulating glycolysis, mitosis, protein biosynthesis, and other cancer related processes [4].

Such biological findings, in combination with the peptide cell-penetrating capacity, made L-2 an attractive lead molecule for further structural optimization. Therefore, the primary sequence of L-2 was modified, including substitution by D-amino acids and N-terminal acetylation [5]. These modifications increased the antitumor effect of the resultant peptide known as CIGB-552 [5, 6].

The CIGB-552* in vitro* antineoplastic effect has been documented by using human cancer cells of different histological origins [5].* In vivo*, a significant reduction in tumor growth after treatment with CIGB-552 was demonstrated in both syngenic murine tumors and patient-derived xenograft models [6]. Additionally to induce apoptosis in the tumor mass, CIGB-552 administration decreased the microvessels' density in the human HT-29 xenograft tumor model suggesting its antiangiogenic effect [6].

Significant advances have been made to discover the functional mediators of CIGB-552 biological response. Copper metabolism (Murr1) domain-containing protein 1 (COMMD1) has been identified as a major target of CIGB-552. Such interaction increased the COMMD1 stability and induced ubiquitination of RelA with subsequent inhibition of the antiapoptotic activity regulated by NF-*κ*B [5]. Nevertheless, the proteome regulated by CIGB-552 can provide new insights to support the antitumor action mechanism of such peptide.

In this work, to identify additional CIGB-552 targets and modulated proteins in tumor cells, two proteomics approaches were used: the chemical proteomics and the expression-based proteomics. The proteomics-derived data suggested that CIGB-552 could be considered as a multitarget drug, which exerts its antitumor effect by modulating proteins related to NF-*κ*B activation, cell cycle regulation, and apoptosis. Network analysis of proteomic results indicates the molecular basis by which the CIGB-552 peptide regulates cancer related processes. The analysis provided here is the starting point for further investigations about the role of CIGB-552 as an anticancer drug.

## 2. Materials and Methods

### 2.1. Peptide Synthesis

CIGB-552 is a cell-penetrating peptide with sequence Ac-HARIK**P**TFRR**L**KWKYKGKFW, where proline and leucine are D-amino acids; and the N-terminal was blocked by acetylation [5]. The CIGB-552 peptide was synthesized on solid phase using the Fmoc strategy; it was purified by reverse phase high performance liquid chromatography (RP-HPLC) to >95% purity on an acetonitrile/H_2_O-trifluoroacetic acid gradient [7] and confirmed by electrospray mass spectrometry (Micromass, UK). For chemical proteomics, the CIGB-552 peptide biotinylated at the N-terminal end (CIGB-552-B) was synthesized using the same procedure.

### 2.2. Cell Culture and Treatments

For proteomic studies two CIGB-552 sensitive cancer cell lines were selected, the larynx carcinoma and colon adenocarcinoma cells, Hep-2 and HT-29, respectively, which were obtained from ATCC (Rockville, MD). Both cell lines were cultured at 37°C and 5% CO_2_ in RPMI 1640 (Life Technologies, USA) supplemented with 10% fetal bovine serum (FBS; PAA, Canada) and 30 *μ*g/mL gentamicin (Sigma, USA).

For expression-based proteomics, 20 × 10^6^ HT-29 cells were seeded in appropriate vessels and incubated for 24 h. Subsequently, the tumor cells were incubated with 150 *μ*mol/L of CIGB-552 during 40 min, 2 h, and 5 h.

### 2.3. Chemical Proteomics

#### 2.3.1. Isolation of Proteins

For chemical proteomics, 20 × 10^6^ Hep-2 cells were seeded in appropriate vessels and cultured for 24 h. Subsequently, Hep-2 cells were collected by centrifugation, washed twice with cold phosphate buffered saline (PBS), and lysed in hypotonic PBS solution (0.1x), containing 1 mM of DDT (Sigma) and complete protease inhibitor (Roche, USA), by three freeze-thaw (37°C) cycles. Cellular lysate was cleared by centrifugation at 12 000 rpm at 4°C for 15 min.

#### 2.3.2. Affinity Purification

The CIGB-552-B peptide was incubated with 50 *μ*L of streptavidin-sepharose matrix (binding capacity: 300 nmol/mL; GE Healthcare, USA) for 1 h. As a control, streptavidin-sepharose matrix without CIGB-552-B peptide was used. Then, 300 *μ*g of total protein from Hep-2 cell lysate, as determined by the Bradford assay (Bio-Rad, USA), was added to each matrix and incubated for 2 h at 4°C. The streptavidin-sepharose matrix was collected by short spin and extensively washed with PBS containing 1 mM DTT and 0.5% NP40. Retained proteins were eluted by heat denaturing with SDS sample buffer.

#### 2.3.3. Sample Preparation and LC-MS/MS Analysis

Affinity-purified proteins were reduced, alkylated with acrylamide, and separated by sodium dodecyl sulfate-polyacrylamide gel electrophoresis (SDS-PAGE). Analytical and preparative gels were silver [8] and Coomassie blue stained, respectively. Each lane of the preparative gel was cut into 12 slices that were faded, washed, and* in situ* digested with sequencing grade trypsin (Promega, USA) during 18 h at 37°C. The resulting peptide mixtures were extracted and desalted with stage tips (Thermo Scientific, USA).

Purified samples were analyzed in an Agilent 1100 series nano LC system (Agilent, USA) coupled online to a QTof-2 tandem mass spectrometer (Micromass, UK). The capillary and cone voltages of the electrospray ionization source were operated with 1.8 kV and 35 V, respectively. Samples were applied at 20 *μ*L/min to a PepMap C18 Precolumn Cartridge (5 mm × 300 *μ*m i.d.) from LC-Packings (USA) and were extensively desalted for 10 min using 0.1% formic acid. The precolumn was switched back onto a C18 capillary column (15 cm × 75 *μ*m i.d., packed with 5 *μ*m, Zorbax 300 SB) and the tryptic peptides were separated using a mobile phase containing 0.1% formic acid, 5–45% acetonitrile gradient over 90 min at 300 nL/min flow rate. Survey scans were acquired during 1 s and a maximum of 4 concurrent MS/MS acquisitions were only triggered for 2+, 3+ charged precursors ions detected at an intensity above a threshold of 15 counts/s. Each MS/MS acquisition was completed and switched back to MS mode when the total ion current fell below a threshold of 2 counts/s or after a maximum of 6 s of continuous acquisition. Data acquisition and processing were performed using MassLynx v3.5 (Micromass, UK).

#### 2.3.4. Protein Identification

Acquired data were searched against the human proteins in the UniProtKB database using MASCOT (version 2.2, Matrix Science, UK) [9]. Search parameters were set to a mass tolerance of 1.2 Da for the precursor ions and 0.6 Da for the fragment ions. One trypsin missed cleavage site was allowed. Propionamide-cysteine and oxidized methionine were set as fixed and variable modifications, respectively. MS/MS spectra of identified proteins with one or two peptides were manually inspected. The identification of a protein or peptide was considered positive using the consensus of several criteria: the peptide score >20, the assignment of four intense consecutive *y*_*n*_′′ fragments ions in the MS/MS spectrum, and the most intense signals which must be explained considering the proposed sequence.

#### 2.3.5. Bioinformatics Analysis

Functional classification of identified proteins was based on the information annotated in the Gene Ontology (GO) database (http://www.geneontology.org). The analysis was performed using the functional enrichment tool GeneCodis (version 3.0) (http://genecodis.cnb.csic.es/) [10]. To identify significant enriched biological processes (*p* values lower than 0.05), the hypergeometric distribution and the false discovery rate (FDR) correction method were computed by GeneCodis, as statistical analysis. Protein complexes associated with the CIGB-552 target profile were identified by using the CORUM database (http://mips.helmholtz-muenchen.de/genre/proj/corum) [11].

A target deconvolution strategy was applied to filter the CIGB-552 target profile. Nonspecific proteins, retained in unloaded streptavidin-sepharose matrix (without CIGB-552-B), were ruled out. Further, proteins reported by Burkard et al. [12] as part of the central proteome were subtracted from the analysis. To identify contaminants or background proteins the potential CIGB-552 target profile was queried against the CRAPome database (http://www.crapome.org). For each protein, the average spectral count was retrieved as a measure of its abundance in affinity purification followed by mass spectrometry (AP-MS) experiments [13].

Functional subnetworks perturbed by CIGB-552 were identified by using drugDisruptNet (http://bioinformatics.cemm.oeaw.ac.at/index.php/downloads-left/87-disruption-of-functional-networks). The impact of CIGB-552 on functional subnetworks was estimated by the score *S*_net_, which was calculated as described by Burkard et al. [14] but with some modifications. In this regard, the affinity of CIGB-552 to its targets (affinity score) was computed irrespective of the protein abundance in a competitive pull-down (fixed to 1 for all potential CIGB-552 targets). Hence, the affinity score was set to be only proportional to the amount of protein pulled down by CIGB-552-B. As a measure of protein abundance, the exponentially modified Protein Abundance Index (emPAI) of each identified protein was retrieved from MASCOT results. Briefly, the emPAI value is calculated as 10^PAI^ − 1 (PAI = observed peptides/observable peptides), being the number of different observed peptides that cover the protein sequence, a rough estimate of the protein amount in mass spectrometric analysis [15].

### 2.4. Expression-Based Proteomics

#### 2.4.1. Isolation of Cytosolic Proteins

HT-29 cells were collected by trypsinization. After washing with PBS, the cells were suspended in 700 *μ*L of the isotonic buffer containing 10 mM Tris-HCl adjusted to pH 7.5, 0.25 M sucrose, 1 mM EGTA, and protease inhibitors. For plasma membrane solubilization Triton X-100 at final concentration of 0.25% was added. After 15 min at 4°C, the cell lysate was centrifuged for 15 min at 12 000 rpm and 4°C. The supernatant containing cytoplasmic proteins was kept at −70°C until subsequent analysis.

#### 2.4.2. Tryptic Digestion and Isobaric Labeling (iTRAQ)

The cytoplasmic protein extracts (120 *μ*g) of CIGB-552-treated and untreated (control) HT-29 cells were precipitated with acetone/TCA and independently dissolved in 20 *μ*L of buffer containing 2 M guanidinium hydrochloride (GuCl) and 500 mM tetraethylammonium bromide (TEAB), pH 8.5. Proteins were reduced using 49 mM tris-2-carboxyethyl phosphine (TCEP) at 60°C for 1 h and alkylated with 84 mM iodoacetamide for 30 min at ambient temperature in the dark. The pool of reduced and S-alkylated proteins was diluted until 50 *μ*L by adding 500 mM TEAB buffer. Proteins were digested with sequencing grade trypsin (Promega, USA) at an enzyme-to-substrate mass ratio of 1 : 10 for 18 h at 37°C.

Tryptic peptides were labeled with iTRAQ reagent according to the manufacturer's protocol (Applied Biosystems, USA). Briefly, each vial of iTRAQ reagent (114–117 tags) was dissolved in 70 *μ*L ethanol by vortexing for 1 min at room temperature. Equal amounts of tryptic peptides derived from different samples were labeled by adding iTRAQ reagent 114 (untreated HT-29 cells, control), iTRAQ reagent 115 (HT-29 cells treated with CIGB-552 for 40 min), iTRAQ reagent 116 (HT-29 cells treated with CIGB-552 for 2 h), and iTRAQ reagent 117 (HT-29 cells treated with CIGB-552 for 5 h). The reaction for iTRAQ labeling was incubated at room temperature for 1 h. Labeled peptides were mixed and dried in a centrifugal evaporator.

#### 2.4.3. LC-MS/MS Analysis

A high pH reversed-phase chromatography step was used to separate the complex mixture of peptides prior to LC-MS/MS analysis. The labeled peptides were resuspended in 500 *μ*L buffer A (0.1% NH_4_OH, pH 10.5) and separated in 24 fractions using a 4.6 mm × 10 cm RP column packed with POROS R2 resin (Applied Biosystems, USA). Peptides were eluted at a flow rate of 0.8 mL/min using a stepwise buffer B (0.1% NH_4_OH, 60% acetonitrile, pH 10.5) gradient. Collected fractions were acidified by adding 200 *μ*L of 5% formic acid, lyophilized, and further dissolved in 200 *μ*L of 0.2% formic acid.

For each fraction, three replicate aliquots of 40 *μ*L were separated in independent LC-MS/MS experiments by reverse phase chromatography. Peptides were separated and analyzed using an Agilent 1100 series nano-LC system (Agilent, USA) coupled online to a QTof-2 orthogonal hybrid tandem mass spectrometer (Micromass, UK) operated as described above. Peptides were eluted at a flow rate of 300 nL/min using a 60 min gradient starting with 5% acetonitrile to 45% acetonitrile with a two-buffer system (Buffer A: 0.2% formic acid; Buffer B: 0.2% formic acid, 80% acetonitrile). Data dependent acquisition MS/MS spectra of the eluted peptides were acquired in three* m/z* ranges (400–600, 590–900, or 890–2000) using the gas-phase fractionation approach [16]. Subsequent analysis proceeded as described above.

#### 2.4.4. Protein Identification

Raw files were processed using MASCOT Distiller software (version 2.3). Peptides were assigned to MS/MS spectra using MASCOT search engine (version 2.2) against the human proteins in the UniProtKB database. The following search parameters were selected: 1.2 Da precursor mass tolerance, 0.6 Da daughter ions mass tolerance, and tryptic search with up to one missed cleavage site. Variable modifications including deamidation of glutamine and asparagine, methionine sulfoxide, and the side reaction of iTRAQ labeling on tyrosine residues were taken into account. Carbamidomethyl cysteine and iTRAQ label on lysine and at the peptide N-terminus were specified as fixed modifications. To accept a peptide hit as positive we considered a false discovery rate (FDR) of 3% based on the target-decoy strategy [17].

Quantification was achieved using ISOTOPICA software [18, 19]. The software enables the relative peptide quantification based on the detailed analysis of the observed isotopic ion distribution. The software calculated the best ratio of the reporter ions (tags 114, 115, 116, and 117) to obtain an* in silico* isotopic ion distribution best matching with the isotopic ion distribution observed experimentally. To evaluate the quality of this adjustment, the software calculates the difference between the area of both* in silico* and experimental isotopic ion distributions, and it is expressed as a GOF coefficient (goodness of fitting). The relative quantifications of peptides with GOF below 0.8 were not considered for further analysis. Additionally all relative quantifications were manually inspected. The relative peptide expression ratios (fold changes) were determined with respect to the control sample (tag 114). The fold changes of all peptides corresponding to the same protein were averaged. The resulting protein fold changes were normalized; population median and standard deviation set the threshold ratio to consider, with a *p* value < 0.1, a protein as differentially expressed in each condition (HT-29 cells treated with CIGB-552 for 40 min, 2 h, or 5 h) with respect to control (untreated HT-29 cells).

#### 2.4.5. Bioinformatics Analysis

The functional classification of differentially expressed proteins and enrichment analysis were performed as described above. For biological network analysis, the Cytoscape software (version 2.8) [20] and accessory applications were used. Protein-protein interaction network was generated using the BisoGenet application (version 1.4) [21] which retrieves information from interaction databases including DIP, BioGrid, HPRD, and BIND. The fold changes of differentially expressed proteins were visualized in the network context using the MultiColoredNodes application [22].

## 3. Results and Discussion

CIGB-552 is a peptide-based drug with antitumor effect and cell-penetrating capacity [4]. The first evidence about CIGB-552 mechanism of action has been already reported [5]. CIGB-552 interacts with COMMD1 protein, increasing its abundance levels. Such evidences were corroborated by Western blot and immunofluorescence detection of COMMD1 in human cancer cells of different histological origins [5]. CIGB-552 induces cellular cytotoxicity in a variety of tumor cell lines [4, 5]. Among them, the Hep-2 larynx carcinoma cell line is highly sensitive to CIGB-552 cytotoxic effect.

**Figure 1:**
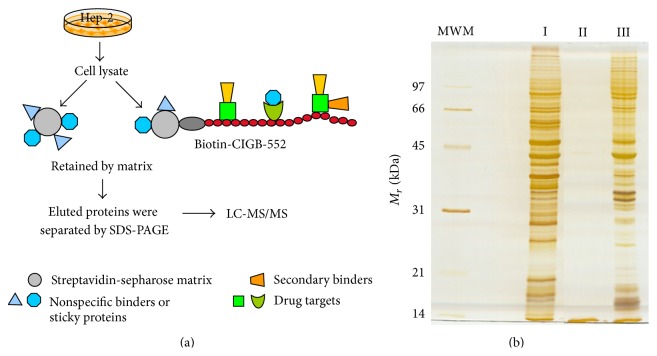
Chemical proteomic approach (a) and results (b). (a) The streptavidin-sepharose (grey) biotinylated CIGB-552 complex retains the drug targets (green) and secondary binders (orange). Nonspecific proteins (blue) stick to the matrix. The retained proteins were eluted, separated by SDS-PAGE, and identified by LC-MS/MS. (b) Proteins eluted from the streptavidin-sepharose matrix were separated by SDS-PAGE. Molecular weight markers (MWM) displayed on the left. Lane I: protein cell extract (Hep-2 cell line); lane II: proteins eluted from the unloaded matrix (without CIGB-552-B); lane III: proteins eluted from the matrix bound to CIGB-552-B.

To identify other proteins that interact with CIGB-552 a chemical proteomic approach was then conducted (Figure 1(a)). PBS-soluble proteins derived from Hep-2 cells were incubated with CIGB-552-B previously bound to the streptavidin-sepharose matrix (Figure 1(a)). As a negative control, the same pool of proteins was also incubated with the unloaded streptavidin-sepharose matrix. CIGB-552 interacting proteins were eluted and analyzed by SDS-PAGE (Figure 1(b)). The electrophoretic pattern of the whole cell extract and CIGB-552-matrix eluted proteins showed appreciable differences in terms of band intensities indicating the potential CIGB-552 target profile. The proteins identified in the negative control correspond to proteins that interact with the matrix, known as either nonspecific binders or sticky proteins.

**Figure 2:**
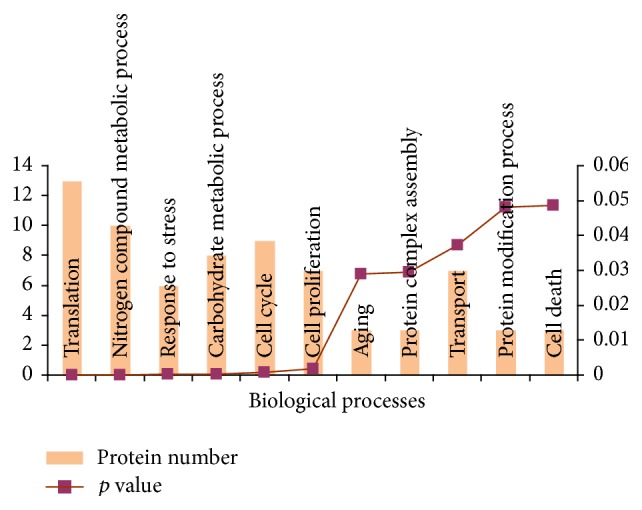
Biological processes significantly represented in the CIGB-552 target profile (161 proteins). Proteins were classified according to annotations of Gene Ontology database; the enrichment analysis was performed using the GeneCodis bioinformatic tool.

A total of 265 proteins were identified by mass spectrometry, of which 104 proteins were also identified as nonspecific binders (see Supplementary Table S1a in the Supplementary Material available online at http://dx.doi.org/10.1155/2015/124082). Therefore, 161 proteins constitute the potential CIGB-552 target profile identified by chemical proteomics. Biological processes related to carbohydrate metabolism, protein modification, and cell cycle are significantly represented on this dataset (Figure 2). Interestingly, such biological processes are also represented in the transcriptomic profile regulated by L-2 peptide in Hep-2 tumor cells [4].

**Figure 3:**
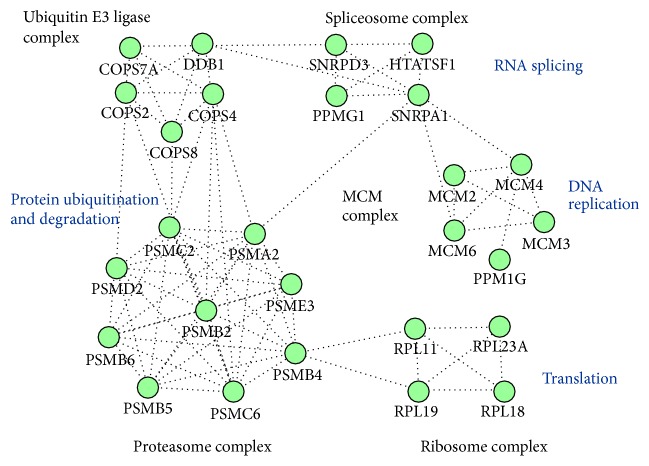
Protein complexes included in the CIGB-552 target profile (161 proteins). Biological processes related to protein complexes are illustrated. The analysis was conducted using CORUM database.

The potential CIGB-552 target profile identified* in vitro* includes five biological complexes (Figure 3). Components of the minichromosome maintenance (MCM) complex were identified as potential CIGB-552 targets. The MCM complex is related to DNA replication and cell cycle regulation. Therefore, the MCM proteins are frequently upregulated in different cancer types including meningioma, lung cancer, and laryngeal carcinoma [23–26]. Four components of the 60S ribosomal large subunit were also identified. Accordingly, translation is a biological process overrepresented in the CIGB-552 target profile (Figure 2). Related to protein modification process, five components of the ubiquitin E3 ligase complex were found. In addition, nine subunits of the proteasome complex were included in the CIGB-552 target profile.

Chemical proteomics do not distinguish between drugs direct and indirect binders. In addition, a high background is typically identified in these experiments [27]. Therefore, the 161 proteins found in the chemical proteomic profile might include nonspecific binders of CIGB-552. Potential solutions to overcome the specificity problem of chemical proteomics are (i) to include unrelated drugs or the matrix itself as negative controls, (ii) to identify and subtract from the analysis the abundant proteins or core proteome, and (iii) to perform a competitive pull-down to rank the most relevant targets [28].

In the present study, besides identifying nonspecific binders, the core proteome was subtracted from the potential CIGB-552 target profile. A set of 1124 proteins previously identified as part of the human central proteome was used [12]. The potential CIGB-552 target profile (161 proteins) includes 106 proteins of the central proteome. The majority of these proteins (91/106) were also reported with an average spectral count greater than two in the CRAPome database (Supplementary Table S1a-b). The spectral count is used as a measure of protein abundance in affinity purification (AP) experiments [13]. Therefore, the CRAPome repository gives information about abundant proteins or common contaminants of AP-MS experiments.

By subtracting the central proteome, 55 proteins were identified as the most probable targets of CIGB-552 (Table 1). This result suggests the potentialities of CIGB-552 as a multitarget drug. In contrast to previously published results [5], the COMMD1 protein was not identified in the chemical proteomic profile, probably due to its low expression levels in tumor cells [29].

The central proteome is mainly enriched in cell vital processes [12]. Consequently, after the central proteome subtraction, biological processes such as translation, transport, response to stress, and cell death are no longer significantly represented in the CIGB-552 chemical proteomic profile. However, proteins related to cell cycle, carbohydrate metabolic process, and signal transduction are corroborated as mediators of the CIGB-552 antitumor activity (Supplementary Table S2a).

Target deconvolution based on the subtractions of sticky and core proteome proteins might remove a real drug target [28]. Nevertheless, this strategy has been previously used to identify the targets of BCR-ABL kinase inhibitor INNO-406 in chronic myeloid leukemia [30]. To overcome the potential disadvantage of removing real targets, a computational strategy was applied for identifying the functional subnetworks perturbed by CIGB-552. Such computational approach, named drugDisruptNet, was presented by Burkard et al. to predict the mechanism and potential side effects of drugs [14]. A functional subnetwork is defined as a connected fraction of the interactome in which all the proteins share the same function [14]. A drug can impact functional subnetworks directly (the drug target is part of the subnetwork) or indirectly (the drug target interacts with the protein subnetwork) [14]. Therefore, even if a CIGB-552 target is removed during the target deconvolution, the remaining potential targets might indicate the functional subnetworks perturbed by the drug.

To identify the functional subnetworks perturbed by CIGB-552, the filtered target profile (55 proteins) was used. As other studies have corroborated the role of COMMD1 in CIGB-552 cytotoxic effect [5], this protein was included in the network analysis. According to drugDisruptNet results (Table 2), different subnetworks related to carcinogenesis were perturbed by CIGB-552, “antiapoptosis” and “negative regulation of cell cycle” among them. These results corroborate the antitumor effect of CIGB-552.

Additionally, “extracellular structure organization” and “response to hypoxia” were modulated by the CIGB-552 target profile (Table 2). Both the composition and organization of the extracellular matrix and hypoxia are microenvironment signals that contribute to metastatic spread of cancer cells [31]. Therefore, CIGB-552 could inhibit metastasis in treated cells.

**Figure 4:**
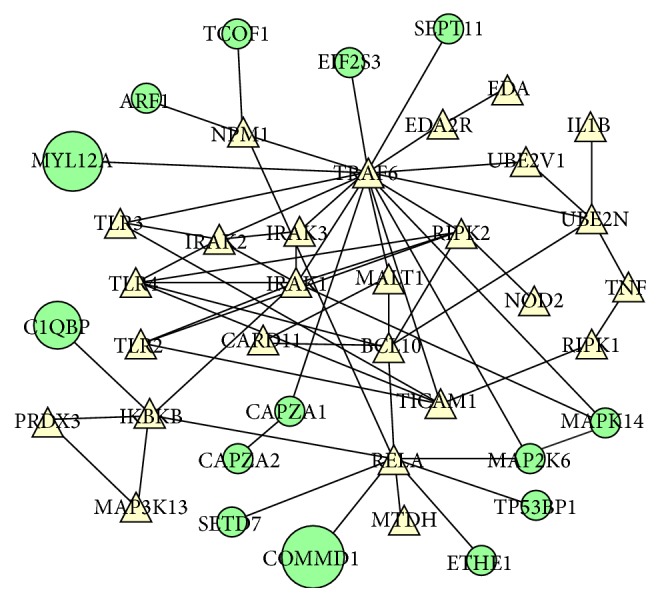
CIGB-552 targets (green nodes) perturb the positive regulation of NF-*κ*B transcription factor activity. In the protein-protein interaction network, the CIGB-552 target profile (green nodes) interferes with proteins that share the same Gene Ontology term (triangular nodes). The node size is proportional to drug affinity, which was calculated based on the abundance of pulled-down proteins according to emPAI values. COMMD1 protein, which is a validated CIGB-552 target, is represented in the functional subnetwork with the maximal node size to illustrate a high drug affinity.

The “positive regulation of NF-*κ*B transcription factor activity” is disrupted by CIGB-552 (Table 2). Although the target profile is not annotated in this signaling pathway, it acts at the periphery of the functional subnetwork (Figure 4). Previous results demonstrated that CIGB-552 treatment upregulates COMMD1 levels [5]. Such event increases RelA ubiquitination and consequently inhibits the NF-*κ*B signaling pathway [5]. In the present study, COMMD1 was not identified in the target profile. However, the “positive regulation of NF-*κ*B transcription factor activity” is disrupted by the CIGB-552 target profile essentially at two network nodes: RELA and TRAF6 (Figure 4).

NF-*κ*B transcription factor is a homo- or heterodimeric complex composed of REL proteins (RelA/p65, RelB, c-Rel, p50/p105, and p52/p100). RelA/p50 appears to be the most common heterodimer. After activation of I-kappa-B kinase complex (IKK), the active NF-*κ*B complex is translocated into the nucleus to regulate gene transcription [32]. NF-*κ*B target genes promote tumor cell proliferation, inhibition of apoptosis, migration, inflammation, and angiogenesis [33]. Therefore, upregulated NF-*κ*B activity has been reported in different tumor types including laryngeal and pancreatic cancers [34–36].

The TNF receptor-associated factor 6 (TRAF6) activates NF-*κ*B [37]. This mechanism depends on TRAF6-mediated TGF-beta-activated kinase 1 (MAP3K7/TAK1) activation, which in turn phosphorylates and activates the I-kappa-B kinase complex (IKK) [38]. MAP3K7/TAK1 also phosphorylates the dual specificity mitogen-activated protein kinase kinase 6 (MAP2K6), which in turn activates the p38 MAPK signaling pathway [37]. In the present study, MAP2K6 and MAPK14 (mitogen-activated protein kinase 14) were identified as potential CIGB-552 targets (Figure 4). MAPK14 is a member of the p38 MAPK family. The p38 MAPKs phosphorylate different substrates, including the nuclear mitogen- and stress-activated protein kinase 1 (RPS6KA5/MSK1). MSK1 phosphorylates RelA and increases the transcriptional activity of NF-*κ*B [39]. Therefore, CIGB-552 could inhibit the NF-*κ*B signaling pathway in tumor cells by blocking the MAP2K6/MAPK14 functions.

Furthermore, persulfide dioxygenase ETHE1 (ETHE1) and lysine N-methyltransferase 7 (SETD7) were identified as potential CIGB-552 targets (Figure 4). Other studies demonstrate that both proteins regulate RelA transcriptional activity. ETHE1 protein promotes accumulation of RelA in the cytoplasm and consequently inhibits the NF-*κ*B transcriptional activity [40]. SETD7 protein monomethylates RelA subunit; this event triggers RelA degradation and downregulates NF-*κ*B target gene expression [41, 42]. The identification of ETHE1 and SETD7 in the target profile suggests that CIGB-552 could exert, by interacting with these proteins, different mechanisms to regulate RelA function.

Interleukin-6 (IL-6) expression is activated by NF-*κ*B transcription factor [43, 44]. The “interleukin-6 production” subnetwork was perturbed by CIGB-552 target profile (Table 2). Therefore, disruption of this subnetwork could be a consequence of inhibiting NF-*κ*B transcriptional activity. Importantly, IL-6 downregulation could be validated as a subrogate marker of CIGB-552 antitumor effect.

CIGB-552 target interactions trigger cellular signaling events that finally accomplish the antitumor response. To investigate these signaling events at the protein level, an expression-based proteomics study was conducted. The majority of proteins identified by chemical proteomics as potential CIGB-552 targets (109/161) are located in the cytosol. Cytoplasmic proteins are also enriched after target deconvolution (36/55) (Supplementary Table S2b-c). Therefore, the cytoplasmic proteome regulated in the presence of 150 *μ*mol/L of CIGB-552 was studied using the HT-29 cell line as target cells. Such peptide dose represents the inhibitory concentration 80 (IC80) for CIGB-552 in HT-29 cells (unpublished results). Confocal microscopy demonstrates that after 10 min of incubation CIGB-552 is able to penetrate the cells [4]. In the present work, the proteomic profile modulated by CIGB-552 was investigated at three incubation times: 40 min, 2 h, and 5 h.

In both proteomic experiments, different tumor cell lines were used to evaluate consistence among results. HT-29 cells, as well as Hep-2 cells, have shown to be sensitive to CIGB-552 anticancer effect [4]. In addition, CIGB-552 was able to elicit significant antitumor activity in both murine CT-26 (colon carcinoma cells) and human HT-29 implanted tumors [6]. Consequently, the differentially expressed proteins in treated HT-29 cells could support the findings of the CIGB-552 target profile identified in total cell extracts from Hep-2 cells.

As a result, 658 protein hits were identified (Supplementary Table S3), of which 72 proteins were differentially modulated in at least one experimental condition (CIGB-552: 40 min, 2 h, and 5 h) with respect to control (Table 3). A total of 68 proteins of the proteomic profile (658 protein hits), including eight differentially modulated proteins in response to CIGB-552 treatment, were also identified by chemical proteomics as potential CIGB-552 targets (Supplementary Table S3).

**Figure 5:**
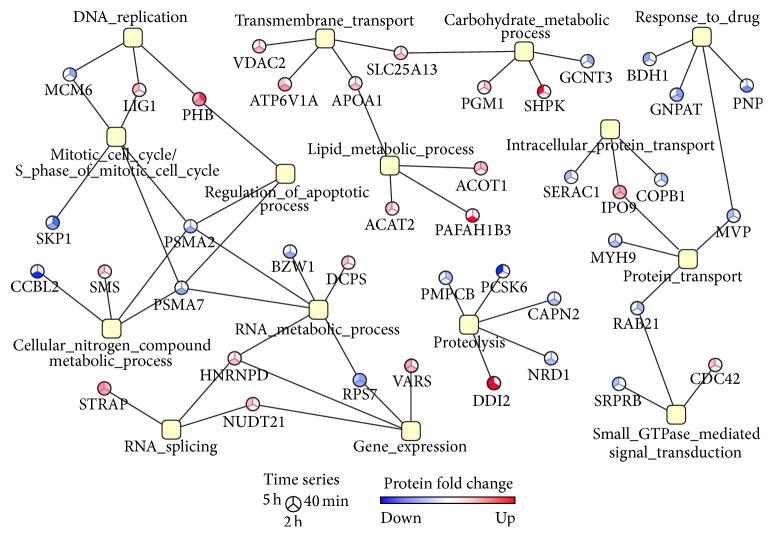
Functional association network that illustrates the biological processes significantly represented (*p* values < 0.05) in the proteomic profile modulated by CIGB-552 on HT-29 cells. Proteins were classified according to annotations of Gene Ontology database; the enrichment analysis was performed using the GeneCodis bioinformatic tool. The color of network nodes representing proteins is proportional to the fold change on CIGB-552-treated cells with respect to control.

Bioinformatics analysis of comparative proteomic data demonstrated the enrichment of biological processes such as gene expression, proteolysis, and response to drug (Figure 5). Importantly, carbohydrate and nitrogen metabolic processes, protein transport, cell cycle, and regulation of apoptosis were found to be significantly represented in chemical and comparative proteomic profiles (Figures 2 and 5). These results demonstrated that even in different cell lines (HT-29, Hep-2) the CIGB-552 antitumor effect is exerted by modulating similar biological processes.

Differentially expressed proteins related to apoptosis and cell cycle regulation support the CIGB-552 antitumor effect. Among them, the DNA replication licensing factor MCM6 (MCM6), the cyclin-G-associated kinase (GAK), and the S-phase kinase-associated protein 1 (SKP1), which function as positive regulators of cell cycle [25, 45, 46], were significantly downregulated after 40 min of CIGB-552 treatment.

**Figure 6:**
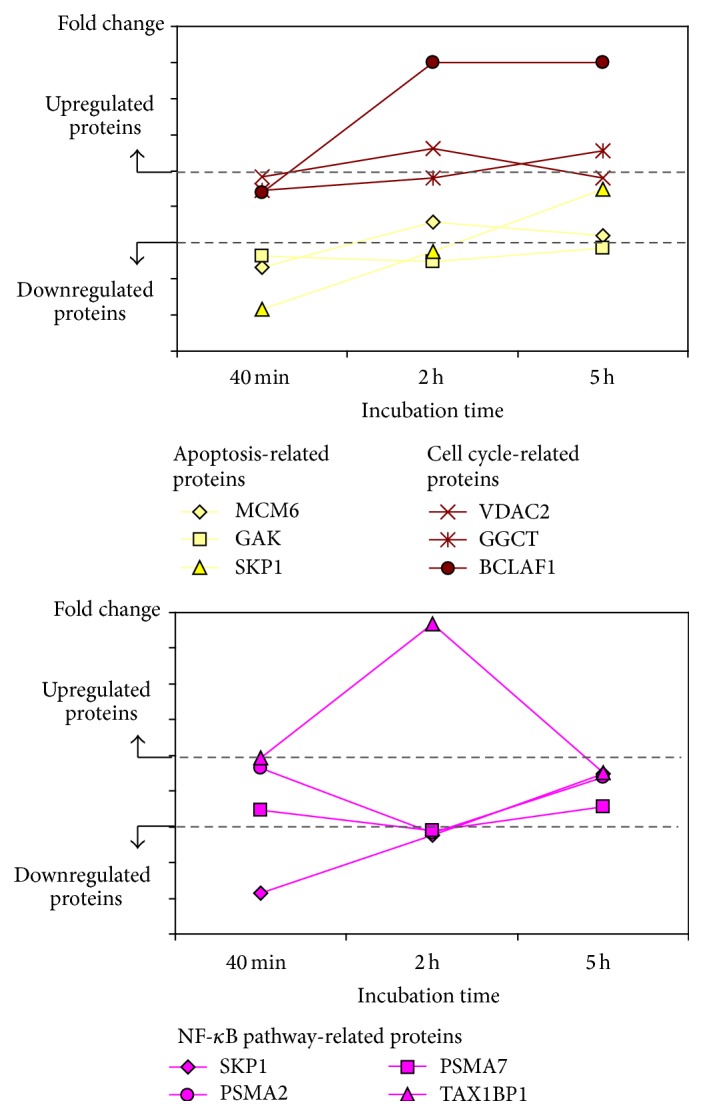
Identification of protein clusters related to cell cycle, apoptosis, and NF-*κ*B signaling, which showed similar trends of differential expression over time.

As expected, on CIGB-552-treated cells, the apoptosis was a much later event than the negative regulation of cell cycle (Figure 6). Only after 2 h, the CIGB-552 treatment increased the expression of proapoptotic proteins. For instance, the voltage-dependent anion-selective channel protein 2 (VDAC2) was significantly upregulated after 2 h of CIGB-552 treatment. This protein is a component of the mitochondrial permeability transition pore complex, which releases apoptogenic factors, such as cytochrome C, during apoptosis [47, 48]. In line with this evidence, the expression of the cytochrome C-releasing factor 21 (GGCT) was increased sequentially on CIGB-552-treated cells, the upregulation being significant after 5 h of treatment. Furthermore, the expression of Bcl-2-associated transcription factor 1 (BCLAF1), a transcriptional repressor that promotes apoptosis [49, 50], was increased after 2 h of treatment with CIGB-552. Concomitant with upregulation of proapoptotic proteins, the expression of the major vault protein (MVP) was significantly decreased on HT-29 cells after 5 h of CIGB-552 treatment (Figure 5). MVP functions as a multidrug resistance factor [51–53]. Therefore, the downregulation of MVP could facilitate the proapoptotic effect in response to CIGB-552 treatment and overcome the chemotherapeutic resistance usually developed by cancer cells.

Most of the proteins related to proteolysis were downregulated by CIGB-552 treatment (Figure 5). Two peptidase M16 family members, which have metalloendopeptidase activity, were identified: the mitochondrial-processing peptidase subunit beta (PMPCB) and nardilysin (NRD1). Particularly, NRD1 protein promotes cell growth and invasion of cancer cells [54, 55]. In the present work, Calpain-2 (CAPN2) and proprotein convertase subtilisin/kexin type 6 (PCSK6) were also identified. Both proteins are proteases related to tumor progression. CAPN2 upregulation increases the invasive potential of different tumor cells; such effect has been associated with secretion of matrix metalloproteinases (MMP-2 and MMP-9) [56–58]. Besides, in the tumor microenvironment, the inhibition of Calpain blocks angiogenesis [59]. PCSK6 substrates include precursors of cancer related proteins such as matrix metalloproteinases and the vascular endothelial growth factor (VEGF) [60]. Therefore, upregulation of PCSK6 increases the invasiveness of carcinoma cells [61–63]. Indeed inhibition of PCSK6 has been proposed as an anticancer therapeutic strategy [64, 65]. Altogether, the concomitant downregulation of NRD1, CAPN2, and PCSK6 suggests that CIGB-552 treatment could inhibit cancer cell invasion, exerting a potential antimetastatic effect. As previously mentioned, the antimetastatic effect of CIGB-552 is also supported by chemical proteomics results. To validate such hypothesis other studies have been conducted.

**Figure 7:**
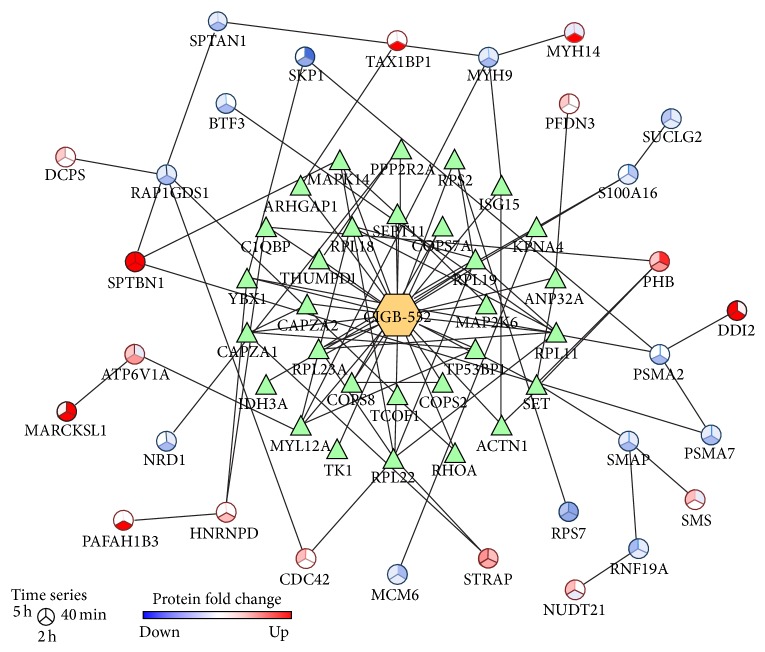
Protein-protein interaction network that represents the intersection between the chemical (triangle nodes) and the comparative (circular nodes) proteomics profiles. The network was represented using BisoGenet plugin, which retrieves interaction data from multiple databases. For network nodes representing differentially expressed proteins, the color is proportional to the fold change on CIGB-552-treated cells with respect to control.

To analyze chemical proteomic data in combination with differentially expressed proteins, an interaction network was represented (Figure 7). According to interaction databases, 18 differentially modulated proteins interact with the potential CIGB-552 target profile (18 proteins). Prohibitin (PHB) interacts with three proteins included in the CIGB-552 target profile (ACTN1, SET, and C1QBP) (Figure 7). The expression of PHB, which is a negative regulator of cell proliferation [66], was increased on CIGB-552-treated cells. Besides, prefoldin subunit 3 (PFDN3), upregulated by CIGB-552 treatment, interacts with the acidic leucine-rich nuclear phosphoprotein 32 family member A (ANP32A) (Figure 7). It is a tumor suppressor protein [67] that was identified as a potential CIGB-552 target. Additionally, PFDN3 binds to von Hippel-Lindau protein (VHL), which regulates ubiquitination and proteasomal degradation of the hypoxia-inducible factor 1-alpha (HIF-*α*) [68]. Functional subnetworks of “negative regulation of cell cycle” and “response to hypoxia” were perturbed by CIGB-552 target profile (Table 2). Accordingly, such effects are supported by the upregulation of PHB and PFDN3 on HT-29 cells treated with CIGB-552.

The intersection between chemical proteomic data and differentially expressed proteins indicates potential mediators of CIGB-552 antitumor effect. The Rho GTPase-activating protein 1 (ARHGAP1) was identified as a potential CIGB-552 target (Figure 7). This protein inactivates signal transduction mediated by Rho-family GTPases, such as RhoA, Rac1, and CDC42 [69, 70]. Accordingly, the functional category of “small GTPase mediated signal transduction” was significantly represented in the proteomic profile modulated by CIGB-552 on HT-29 cells (Figure 5). In addition, ARHGAP1 interacts with Tax1-binding protein 1 (TAX1BP1) (Figure 7) [71]. The expression of TAX1BP1 was increased at 2 h of CIGB-552 treatment (Table 3). TAX1BP1, in conjugation with the zinc finger protein A20, the E3 ubiquitin-protein ligase Itch, and RING finger protein 11, constitutes the A20 ubiquitin-editing protein complex [72]. Downstream to Toll-like receptor 4 (TLR4) and tumor necrosis factor receptor 1 (TNFR1) the A20 complex inactivates TRAF6 and RIP1, repressing the NF-*κ*B signaling pathway [73, 74]. Furthermore, in the present work, several potential CIGB-552 targets interact with TRAF6 (Figure 4). Altogether, these results suggest that CIGB-552 could inhibit the TRAF6/NF-*κ*B axis.

Other proteins related to NF-*κ*B signaling pathway were also modulated on HT-29 cells by CIGB-552. The S-phase kinase-associated protein 1 (SKP1) was downregulated at 40 min and 2 h after CIGB-552 treatment (Figure 7). This protein is an essential component of the SCF (SKP1-CUL1-F-box protein) E3 ubiquitin ligase complex, which mediates the ubiquitination of proteins involved in cell cycle progression, signal transduction, and transcription [75]. As part of SCF complex, the F-box/WD repeat-containing protein 1A (BTRC) ubiquitinates I*κ*B leading to its proteasomal degradation and consequently NF-*κ*B activation [76]. In the present work, the F-box protein BTRC was not identified. However, by decreasing the expression of SKP1, CIGB-552 could compromise the function of SCF complex. SKP1 is a proteasome interacting protein (Figure 7) [77]. The expression of two proteasome subunits (PSMA2, PSMA7) was decreased at 2 h of CIGB-552 treatment (Figure 7). Besides, several components of the proteasome complex, including the subunit PSMA2, were identified by chemical proteomics as potential CIGB-552 targets (Figure 3). Interestingly, modulation of SKP1 and proteasome subunits is concomitant with TAX1BP1 upregulation (Figure 6). These results suggest that CIGB-552 potentiates, at 2 h following treatment, the inhibition of NF-*κ*B signaling pathway. Such effect is also supported by chemical proteomic results, as the “positive regulation of NF-*κ*B transcription factor activity” is a functional subnetwork disrupted by the CIGB-552 target profile (Figure 4).

**Figure 8:**
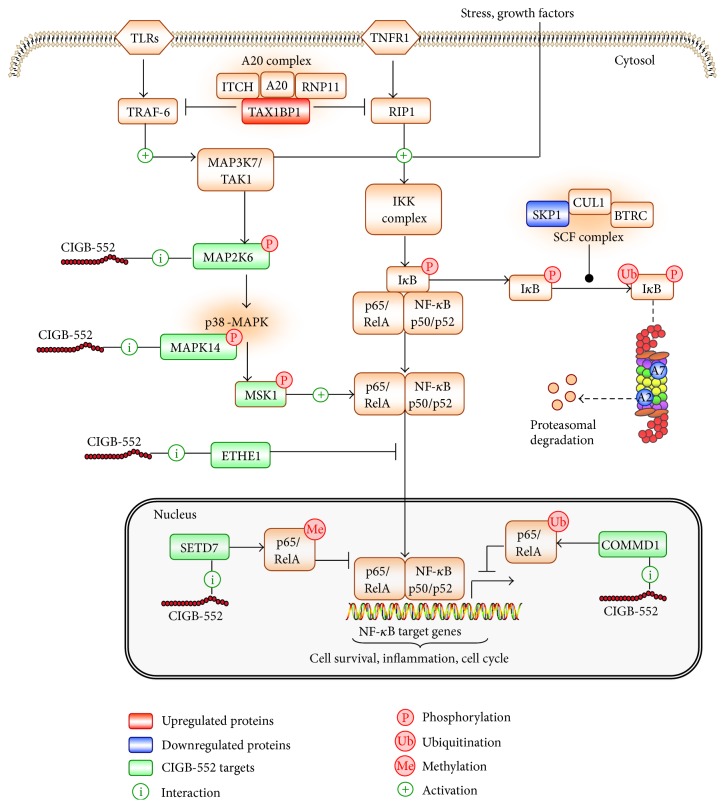
Proposed mechanism for NF-*κ*B signaling inhibition mediated by the antitumor peptide CIGB-552. Different proteins, which regulate NF-*κ*B pathway, were differentially modulated by CIGB-552 treatment (TAX1BP1, SKP1, PSMA2/A2, and PSMA7/A7). In addition, the CIGB-552 target profile includes proteins that regulate RelA function (ETHE1, SETD7, and MAPK signaling: MAP2K6, MAPK14). Such interactions ultimately could repress the transcription of NF-*κ*B target genes. Altogether, by modulating the function of these proteins CIGB-552 could inhibit cell proliferation and induces apoptosis of tumor cells.

A potential mechanism of NF-*κ*B inhibition by CIGB-552 was proposed based on both chemical and comparative proteomic profiles (Figure 8). Previous results demonstrated that CIGB-552 interacts with COMMD1 triggering the RelA ubiquitination [5]. Other potential CIGB-552 interactors include the proteins ETHE1 and SETD7. ETHE1 and SETD7, as well as COMMD1, regulate the function of RelA [40–42]. Therefore, CIGB-552 by interacting with ETHE1 and SETD7 could repress the transcription of NF-*κ*B target genes (Figure 8). As the MAPK cascade increases the transcriptional activity of RelA [39], interaction between CIGB-552 and MAPKs proteins (MAP2K6, MAPK14) could also inhibit the NF-*κ*B signaling pathway (Figure 8). Finally, the upregulation of TAX1BP1 and the downregulation of SKP1 induced by CIGB-552 are of great relevance. From a mechanistic view, the modulation of both proteins by CIGB-552 supports the inhibition of NF-*κ*B signaling pathway (Figure 8).

## 4. Conclusions

This study explored at the proteomic level the CIGB-552 antitumor mechanism of action. The CIGB-552 target profile was investigated in Hep-2 cells using a chemical proteomic approach. In total, 161 proteins were identified as potential CIGB-552 targets. This target profile was reduced to 55 proteins by a target deconvolution strategy. According to chemical proteomic results, CIGB-552 could be a multitarget drug. Downstream to drug-target interactions proteins that mediate cellular response to CIGB-552 treatment were identified by a comparative proteomic experiment including time series (40 min, 2 h, and 5 h). The proteomic profile modulated by CIGB-552 on HT-29 cells includes 72 differentially expressed proteins. The results of both experimental approaches, chemical and expression-based proteomics, were highly consistent. Proteins related to NF-*κ*B signal transduction were identified as potential CIGB-552 targets and were significantly modulated by CIGB-552 treatment. Such findings are in line with previous results demonstrating that CIGB-552 blocks NF-*κ*B signaling by upregulation of COMMD1 and consequently ubiquitination of RelA. The proteomic data revealed new mediators for NF-*κ*B inhibition in response to CIGB-552 treatment. Proteins related to cell proliferation and apoptosis were identified by chemical and expression-based proteomics. These differentially expressed proteins could represent subrogate biomarkers of the CIGB-552 effect on tumor cells. The negative regulation of cell cycle and promotion of apoptosis seem to be early and late events, respectively, triggered by treatment with CIGB-552. Proteins related to cell invasion were differentially modulated on CIGB-552-treated cells. This biological process was also represented in the functional subnetworks perturbed by the potential CIGB-552 targets. Therefore, an antimetastatic effect could be expected as a consequence of CIGB-552 treatment. Further experiments are required to corroborate the role of identified proteins on mediating the anticancer effect of CIGB-552. Overall, the current study contributes to a better understanding of the CIGB-552 potentialities for cancer therapy.

## Supplementary Material

Supplementary Table S1a contains the proteins purified on the CIGB-552 streptavidin sepharose matrix from the Hep-2 cell lysate.
Supplementary Table S1b contains the CRAPome information about the central proteome proteins which were identified as part of the CIGB-552 target profile.
Supplementary Table S2a contains biological processes enriched in the CIGB-552 target profile after target deconvolution.
Supplementary Table S2b contains cellular components enriched in the CIGB-552 target profile.
Supplementary Table S2c contains cellular components enriched in the CIGB-552 target profile after target deconvolution.
Supplementary Table S3 contains the proteomic profile modulated by CIGB-552 in HT-29 cells.

## Figures and Tables

**Table 1:** Proteins identified by chemical proteomics as potential CIGB-552 targets.

UniProt ACC^a^	Description	Gene symbol^b^	Score^c^	emPAI^d^
O95218	Zinc finger Ran-binding domain-containing protein 2	ZRANB2	39	0.21
Q9NX65	Zinc finger and SCAN domain-containing protein 32	ZSCAN32	26	0.05
Q9UBQ0	Vacuolar protein sorting-associated protein 29	VPS29	56	0.66
Q14376	UDP-glucose 4-epimerase	GALE	38	0.1
Q12888	Tumor suppressor p53-binding protein 1	TP53BP1	28	0.02
O14773	Tripeptidyl-peptidase 1	TPP1	76	0.06
Q13428	Treacle protein	TCOF1	27	0.02
P04183	Thymidine kinase, cytosolic	TK1	24	0.15
Q9NXG2	THUMP domain-containing protein 1	THUMPD1	58	0.09
Q8WW59	SPRY domain-containing protein 4	SPRYD4	39	0.16
P63151	Serine/threonine-protein phosphatase 2A 55 kDa regulatory subunit B alpha isoform	PPP2R2A	87	0.07
Q9NVA2	Septin-11	SEPT11	29	0.08
O00764	Pyridoxal kinase	PDXK	59	0.11
Q16740	ATP-dependent Clp protease proteolytic subunit, mitochondrial	CLPP	67	0.26
Q9Y4X5	E3 ubiquitin-protein ligase ARIH1	ARIH1	29	0.06
O15460	Prolyl 4-hydroxylase subunit alpha-2	P4HA2	41	0.12
P40261	Nicotinamide N-methyltransferase	NNMT	46	0.27
P19105	Myosin regulatory light chain 12A	MYL12A	200	2.45
Q16539	Mitogen-activated protein kinase 14	MAPK14	82	0.19
Q9UNF1	Melanoma-associated antigen D2	MAGED2	238	0.18
P50213	Isocitrate dehydrogenase [NAD] subunit alpha, mitochondrial	IDH3A	64	0.2
P05161	Ubiquitin-like protein ISG15	ISG15	34	0.21
O00629	Importin subunit alpha-3	KPNA4	49	0.06
Q8WTS6	Histone-lysine N-methyltransferase SETD7	SETD7	42	0.09
P06737	Glycogen phosphorylase, liver form	PYGL	40	0.04
P30712	Glutathione S-transferase theta-2	GSTT2	96	0.14
P47755	F-actin-capping protein subunit alpha-2	CAPZA2	109	0.38
P52907	F-actin-capping protein subunit alpha-1	CAPZA1	118	0.54
P41091	Eukaryotic translation initiation factor 2 subunit 3	EIF2S3	43	0.07
O95571	Persulfide dioxygenase ETHE1, mitochondrial	ETHE1	35	0.13
Q9BVJ7	Dual specificity protein phosphatase 23	DUSP23	50	0.23
P52564	Dual specificity mitogen-activated protein kinase kinase 6	MAP2K6	29	0.1
Q14691	DNA replication complex GINS protein PSF1	GINS1	24	0.16
Q3LXA3	Bifunctional ATP-dependent dihydroxyacetone kinase/FAD-AMP lyase (cyclizing)	DAK	45	0.06
P21291	Cysteine and glycine-rich protein 1	CSRP1	47	0.39
Q99627	COP9 signalosome complex subunit 8	COPS8	69	0.35
Q9UBW8	COP9 signalosome complex subunit 7a	COPS7A	46	0.12
P61201	COP9 signalosome complex subunit 2	COPS2	136	0.23
Q07021	Complement component 1 Q subcomponent-binding protein, mitochondrial	C1QBP	648	1.74
Q9NT62	Ubiquitin-like-conjugating enzyme ATG3	ATG3	58	0.1
Q9BZZ5	Apoptosis inhibitor 5	API5	173	0.13
P12814	Alpha-actinin-1	ACTN1	1345	1.76
P11766	Alcohol dehydrogenase class 3	ADH5	78	0.19
P36404	ADP-ribosylation factor-like protein 2	ARL2	38	0.4
P40616	ADP-ribosylation factor-like protein 1	ARL1	39	0.19
P84077	ADP-ribosylation factor 1	ARF1	99	0.66
P00813	Adenosine deaminase	ADA	35	0.09
Q9BTT0	Acidic leucine-rich nuclear phosphoprotein 32 family member E	ANP32E	169	0.41
P84098	60S ribosomal protein L19	RPL19	38	0.16
P61586	Transforming protein RhoA	RHOA	32	0.17
Q07960	Rho GTPase-activating protein 1	ARHGAP1	63	0.07
Q14657	EKC/KEOPS complex subunit LAGE3	LAGE3	34	0.26
Q96IU4	Alpha/beta hydrolase domain-containing protein 14B	ABHD14B	67	0.17
P39687	Acidic leucine-rich nuclear phosphoprotein 32 family member A	ANP32A	286	1.38
O75607	Nucleoplasmin-3	NPM3	33	0.2

^a^UniProtKB/Swiss-Prot entry accession number (http://www.uniprot.org/).

^b^Recommended gene name (official gene symbol) as provided by UniProtKB/Swiss-Prot.

^c^MASCOT protein score.

^d^emPAI: exponentially modified Protein Abundance Index.

**Table 2:** Functional subnetworks perturbed by CIGB-552.

Perturbed functional subnetworks	GO id.^a^	Nodes	Targets^b^	Score *S*_net_^c^
Insulin receptor signaling pathway	GO:0008286	57	15	0.175
Positive regulation of biosynthetic process	GO:0009891	73	19	0.173
Positive regulation of NF-*κ*B transcription factor activity	GO:0051092	40	14	0.165
Protein autoprocessing	GO:0016540	67	14	0.165
Extracellular structure organization	GO:0043062	66	13	0.143
Mitochondrion organization	GO:0007005	68	17	0.136
Antiapoptosis	GO:0006916	178	26	0.112
Interleukin-6 production	GO:0032635	21	8	0.105
Response to hypoxia	GO:0001666	68	14	0.103
Negative regulation of cell cycle	GO:0045786	37	8	0.076

^a^GO id.: Gene Ontology identifier.

^b^Number of proteins related to the functional subnetwork and identified as potential CIGB-552 targets.

^c^ *S*_net_: score calculated by drugDisruptNet, predicting the impact of CIGB-552 over the functional subnetwork.

**Table 3:** Differentially expressed proteins in HT-29 cells treated with CIGB-552.

UniProt ACC^a^	Description	Gene symbol^b^	Score^c^	Fold change^d^		
				40 min (2.6)	2 h (2.6)	5 h (2.4)
*Downregulated proteins*						
**Q14566**	DNA replication licensing factor MCM6	MCM6	76.01	**−4.2**	−1.1	−2
Q14764	Major vault protein	MVP	88.9	−1.2	−1.9	**−2.6**
**P35579**	Myosin-9	MYH9	166.85	−1.7	**−2.9**	−1.6
P61160	Actin-related protein 2	ACTR2	60.97	**−2.6**	−1.4	−1.8
Q02338	D-beta-hydroxybutyrate dehydrogenase, mitochondrial	BDH1	71.17	−1.7	−1.4	**−2.6**
P20290	Transcription factor BTF3	BTF3	62.09	1.2	**−2.9**	−1.5
Q7L1Q6	Basic leucine zipper and W2 domain-containing protein 1	BZW1	95.07	−1.3	**−3.8**	−2.3
P17655	Calpain-2 catalytic subunit	CAPN2	101.09	−1.3	**−3.1**	−1.9
A8K010	Putative uncharacterized protein C6orf176	C6orf176	33.72	−1.1	**−3.3**	−1.6
P53618	Coatomer subunit beta	COPB1	64.4	**−2.9**	−1.5	−1.4
O60888	Protein CutA	CUTA	62.42	1.2	−2.2	**−2.9**
O14976	Cyclin-G-associated kinase	GAK	47.67	**−3.4**	**−3.8**	**−2.9**
O95395	Beta-1,3-galactosyl-O-glycosyl-glycoprotein beta-1,6-N-acetylglucosaminyltransferase 3	GCNT3	59.62	**−3.2**	−1.2	−1.6
P52306	Rap1 GTPase-GDP dissociation stimulator 1	RAP1GDS1	66.74	−1.2	**−3**	−1.1
O15228	Dihydroxyacetone phosphate acyltransferase	GNPAT	46.58	**−4.5**	**−3.1**	−1.9
Q86Y56	HEAT repeat-containing protein 2	HEATR2	63.07	−1.5	**−5**	−1.4
Q6YP21	Kynurenine-oxoglutarate transaminase 3	CCBL2	39.9	1.7	**OFF**	1.5
P43243	Matrin-3	MATR3	269.16	1	−1.2	**−4.5**
O75439	Mitochondrial-processing peptidase subunit beta	PMPCB	99.73	**−2.9**	1.2	−1.1
**O43847**	Nardilysin	NRD1	41.16	−1.3	**−2.9**	1.6
O15270	Serine palmitoyltransferase 2	SPTLC2	62.71	**−3.2**	−2	**−2.5**
P29122	Proprotein convertase subtilisin/kexin type 6	PCSK6	30.52	−1.3	−1.5	**−16.7**
P00491	Purine nucleoside phosphorylase	PNP	91.96	−2	**−5.6**	1.7
**P25787**	Proteasome subunit alpha type 2	PSMA2	75.42	1.6	**−2.9**	1
O14818	Proteasome subunit alpha type 7	PSMA7	103.36	−1.3	**−2.8**	−1.1
Q13813	Spectrin alpha chain, nonerythrocytic 1	SPTAN1	112.76	−1.6	**−3.1**	−1.6
Q9UL25	Ras-related protein Rab-21	RAB21	129.33	**−2.6**	1.1	−1.1
Q9NV58	E3 ubiquitin-protein ligase RNF19A	RNF19A	31.17	−1.4	−1.8	**−4.8**
P62081	40S ribosomal protein S7	RPS7	65.3	**−4.2**	**−3.8**	**−2.6**
Q96FQ6	Protein S100-A16	S100A16	78	**−2.6**	−1.4	1
P63208	S-phase kinase-associated protein 1	SKP1	72.23	**−7.1**	**−3.1**	1.2
O00193	Small acidic protein	SMAP	74.25	−1.5	**−2.6**	−1.3
Q96JX3	Protein SERAC1	SERAC1	32.13	−1.1	1.3	**−2.5**
Q9Y5M8	Signal recognition particle receptor subunit beta	SRPRB	54.95	−1.7	−1.1	**−2.9**
Q96I99	Succinyl-CoA ligase [GDP-forming] subunit beta, mitochondrial	SUCLG2	85.7	−1.5	−2.4	**−2.4**

*Upregulated proteins*						
O75223	Gamma-glutamylcyclotransferase	GGCT	37.33	1.1	2	**3.8**
P61758	Prefoldin subunit 3	VBP1	152.64	2.1	2.2	**2.5**
Q9BWD1	Acetyl-CoA acetyltransferase, cytosolic	ACAT2	69.2	2.5	2	**3.2**
Q86TX2	Acyl-coenzyme A thioesterase 1	ACOT1	73.32	**2.8**	1.6	**2.4**
P02647	Apolipoprotein A-I	APOA1	76.16	1.9	**2.7**	−1.1
O00192	Armadillo repeat protein deleted in velocardiofacial syndrome	ARVCF	46	**ON**	**ON**	**ON**
Q9NYF8	Bcl-2-associated transcription factor 1	BCLAF1	86.18	1	**ON**	**ON**
O14523	C2 domain-containing protein 2-like	C2CD2L	31.3	**2.9**	1.7	2
P60953	Cell division control protein 42 homolog	CDC42	52.58	2.3	1.5	**3.1**
Q9UJS0	Calcium-binding mitochondrial carrier protein Aralar2	SLC25A13	57.95	2.4	**3.4**	1.9
O43809	Cleavage and polyadenylation specificity factor subunit 5	NUDT21	70.35	1.2	−1.7	**2.5**
**Q96C86**	Scavenger mRNA-decapping enzyme DcpS	DCPS	70.59	1.5	2.1	**2.4**
Q5TDH0	Protein DDI1 homolog 2	DDI2	31.55	1	**ON**	**ON**
P18858	DNA ligase 1	LIG1	49.53	2.3	2.2	**2.6**
Q5JZY3	Ephrin type A receptor 10	EPHA10	37.22	**ON**	**ON**	**ON**
O76003	Glutaredoxin-3	GLRX3	50.31	**3.2**	1.8	**2.4**
Q14103	Heterogeneous nuclear ribonucleoprotein D0	HNRNPD	138.39	1.4	**3**	1.4
**Q96P70**	Importin-9	IPO9	88.04	**3.9**	**2.9**	**2.9**
**P33176**	Kinesin-1 heavy chain	KIF5B	102.25	**4.3**	**3.6**	**5.1**
P49006	MARCKS-related protein	MARCKSL1	112.06	**ON**	**ON**	1
Q9H3R2	Mucin-13	MUC13	68.36	1.8	**4.2**	1.9
Q7Z406	Myosin-14	MYH14	90.59	−1.1	**9.7**	−1.9
Q9NX40	OCIA domain-containing protein 1	OCIAD1	87.8	**2.6**	**2.9**	2.3
Q15102	Platelet-activating factor acetylhydrolase IB subunit gamma	PAFAH1B3	84.5	2.4	**62.4**	1.6
O43252	Bifunctional 3′-phosphoadenosine 5′-phosphosulfate synthetase 1	PAPSS1	51.9	1	1	**ON**
P36871	Phosphoglucomutase-1	PGM1	105.24	**3.3**	−2.2	2.2
P35232	Prohibitin	PHB	90.52	**8.4**	**4.7**	**3.2**
Q9UHJ6	Sedoheptulokinase	SHPK	59.09	1	1	**ON**
P52788	Spermine synthase	SMS	60.56	−1.2	2.1	**3.2**
Q01082	Spectrin beta chain, brain 1	SPTBN1	63.84	**ON**	**ON**	**ON**
Q9Y3F4	Serine-threonine kinase receptor-associated protein	STRAP	80.3	**2.9**	**3.7**	**4.5**
**P26640**	Valyl-tRNA synthetase	VARS	54.77	1.7	**3**	**3.5**
Q86VP1	Tax1-binding protein 1	TAX1BP1	31.29	2.3	**11.7**	1.2
Q9HD45	Transmembrane 9 superfamily member 3	TM9SF3	71.54	1.5	**3.1**	−1.8
P55327	Tumor protein D52	TPD52	64.69	**5.9**	1.8	1.8
P38606	V-type proton ATPase catalytic subunit A	ATP6V1A	66.31	−1.2	**4.4**	**2.8**
P45880	Voltage-dependent anion-selective channel protein 2	VDAC2	59.14	2.1	**4**	2

^a^UniProtKB/Swiss-Prot entry accession number (http://www.uniprot.org/). The UniProt accession numbers highlighted in bold indicate those proteins (8) that were also identified by chemical proteomics.

^b^Recommended gene name (official gene symbol) as provided by UniProtKB/Swiss-Prot.

^c^MASCOT protein score.

^d^Fold change of differentially regulated proteins after 40 min, 2 h, and 5 h of treatment with CIGB-552 [(−) downregulated]. For each incubation time with CIGB-552, is given the fold change threshold to consider a protein significantly up- or downregulated. The fold changes that have an absolute value higher than the threshold are highlighted in boldface.
